# Anti-citrullinated protein antibodies and bone loss in patients with early arthritis: comment on the article “Anti-citrullinated protein antibodies and high levels of rheumatoid factor are associated with systemic bone loss in patients with early untreated rheumatoid arthritis” by Bugatti et al.

**DOI:** 10.1186/s13075-017-1362-5

**Published:** 2017-07-03

**Authors:** Santos Castañeda, Irene Llorente, Rosario García-Vicuña, Isidoro González-Álvaro

**Affiliations:** Rheumatology Division, Hospital de La Princesa, IIS-IP, Universidad Autónoma, c/Diego de León 62, 28006 Madrid, Spain

We read with great interest the article about the relationship between anti-citrullinated protein antibodies (ACPA), rheumatoid factor (RF) and systemic bone loss in 155 patients with early untreated rheumatoid arthritis (RA), published recently in *Arthritis Research & Therapy* [1]. The work is really interesting since it shows the association of ACPA with low bone mass in both lumbar spine and total hip in patients with early RA [1]. These findings are supported by recent investigations demonstrating the expression of citrullinated antigens on the surface of osteoclastic linage cells, which converts these cells into prime targets for circulating ACPA [2, 3].

In this regard, we want to reinforce these findings with data from the baseline visit of 578 patients from our early arthritis (EA) register, the “Princesa Early Arthritis Register Longitudinal (PEARL)”. Briefly, our data show similar results to those described in Bugatti et al.’s work [1], with the difference that we didn’t find any association with RF although our population is larger than theirs and includes early RA and undifferentiated arthritis, strengthening the importance of ACPA in the pathogenesis of bone destruction in inflammatory processes [4]. It is important to emphasize that our series is probably very similar genetically to Bugatti et al.’s [1], with comparable demographic and intrinsic characteristics of the disease [4].

Furthermore, in our work there were no differences in juxtaarticular bone mineral density (BMD) at the metacarpophalangeal joints between ACPA-positive and ACPA-negative patients, suggesting that active inflammation was not the main mechanism of systemic bone loss in early stages of the disease [4].

Additionally, we also analyzed the levels of anti-mutated citrullinated vimentin IgG antibodies, as they could develop a greater pathogenic role [2]. However, their predictive value for bone loss did not improve the results obtained with ACPA, despite the good correlation between the serum levels of both autoantibodies [4].

At the time we submitted our data for publication, no previous publications had described the association of ACPA and BMD in patients with EA. In addition, considering the scarce current evidence of the effect of ACPA on systemic BMD, we believe that the fact that two independent groups report similar findings in such a close time frame make these results more reliable and challenging.

Recently, Orsolini et al. [5] published similar data in a cohort of patients with established RA, demonstrating a titer-dependent effect of ACPA on systemic bone mass, especially at femoral sites (cortical bone), supporting the above-mentioned findings.

## Abbreviations

ACPA: Anti-citrullinated protein antibodies
BMD: Bone mineral density
EA: Early arthritis
PEARL: Princesa Early Arthritis Register Longitudinal
RA: Rheumatoid arthritis
RF: Rheumatoid factor

## References

[1] Bugatti S, Bogliolo L, Vitolo B, Manzo A, Montecucco C, Caporali R (2016). Anti-citrullinated protein antibodies and high levels of rheumatoid factor are associated with systemic bone loss in patients with early untreated rheumatoid arthritis. Arthritis Res Ther..

[2] Harre U, Georgess D, Bang H, Bozec A, Axmann R, Ossipova E (2012). Induction of osteoclastogenesis and bone loss by human autoantibodies against citrullinated vimentin. J Clin Invest..

[3] Malmström V, Catrina AI, Klareskog L (2017). The immunopathogenesis of seropositive rheumatoid arthritis: from triggering to targeting. Nat Rev Immunol..

[4] Llorente I, Merino L, Ortiz AM, Escolano E, González-Ortega S, García-Vicuña R, et al. Anti-citrullinated protein antibodies are associated with decreased bone mineral density: baseline data from a register of early arthritis patients. Rheumatol Int. 2017;37:799-806. doi:10.1007/s00296-017-3674-9 [Epub ahead of print]. 10.1007/s00296-017-3674-9 PMC5397447 28243799

[5] Orsolini G, Caimmi C, Viapiana O, Idolazzi L, Fracassi E, Gatti D, et al. Titer-dependent effect of anti-citrullinated protein antibodies on systemic bone mass in rheumatoid arthritis patients. Calcif Tissue Int. 2017;101:17-23. doi:10.1007/s00223-017-0253-8 [Epub ahead of print]. 10.1007/s00223-017-0253-8 28246933

[6] Bugatti S, Bogliolo L, Montecucco C, Manzo A (2016). B cell autoimmunity and bone damage in rheumatoid arthritis. Reumatismo..

[7] Kleyer A, Finzel S, Rech J, Manger B, Krieter M, Faustini F (2014). Bone loss before the clinical onset of rheumatoid arthritis in subjects with anti citrullinated protein antibodies. Ann Rheum Dis..

