# Supplementary material for: Anti-citrullinated protein antibodies and bone loss in patients with early arthritis: comment on the article “Anti-citrullinated protein antibodies and high levels of rheumatoid factor are associated with systemic bone loss in patients with early untreated rheumatoid arthritis” by Bugatti et al
Source: Arthritis Res Ther. 2017 Jul 3;19:152. doi: 10.1186/s13075-017-1362-5 (PMC5496424; doi:10.1186/s13075-017-1362-5)
Supplement: Related texts

# Reply: Authors’ response

**Authors:** Serena Bugatti, Carlomaurizio Montecucco

We are grateful to Castañeda and colleagues for their interest in our paper recently published in *Arthritis Research & Therapy* [1]. Increasing evidence links the presence and the characteristic of autoantibodies to relevant pathophysiological processes in rheumatoid arthritis (RA), also offering a causal explanation of the long-known epidemiological association between seropositivity and worse outcomes [6]. In this direction, the discovery of a direct pro-osteoclastic effect of anti-citrullinated peptide antibodies (ACPA) [2] has revolutionized the assumption that tissue damage in RA depends exclusively on the action of pro-inflammatory cytokines. Following that publication, clinical scientists have put great effort into exploring the early effects of autoantibodies on bone. Individuals with ACPA but no evidence of synovitis show cortical bone changes at the metacarpophalangeal joints [7], and, similarly, we have found that patients with a very short history of RA present reduced systemic bone mineral density only in association with RA-specific autoantibodies [1]. Nicely, in their letter Castañeda and colleagues report similar findings obtained in a large cohort of patients with early arthritis, reinforcing the concept that local and generalized bone remodeling in RA may be at least in part disconnected from classic inflammatory pathways, and that strategies aimed at halting/preventing bone loss should include close monitoring of autoantibody-positive subjects beyond the control of disease activity.

Research on the causal relationship between autoantibodies and RA pathology is in its infancy, and many aspects need further clarification. Among these, the role of rheumatoid factor (RF) remains debated. Whilst we and others have reported a potential additive effect of RF on the background of ACPA-positivity, Castañeda et al. failed to find an association between RF and systemic bone loss. Compared to ACPA, RF testing shows much higher variability in the clinical setting due to greater fluctuations in levels in the same patient over time and to larger differences in test characteristics among different laboratories. These issues, along with the inclusion of non-RA patients in Castañeda et al.’s series, may partially explain the conflicting results. However, the pentameric IgM RF activates complement and enhances, in a dose-dependent way, the pro-inflammatory effect of ACPA on macrophages in vitro, and several clinical observations link high RF levels with more active and more destructive disease [6]. Further research in this field is welcome, as it would translate into better knowledge on the role of autoantibodies as biomarkers and possible therapeutic targets in RA.
